# Surface drag reduction and flow separation control in pelagic vertebrates, with implications for interpreting scale morphologies in fossil taxa

**DOI:** 10.1098/rsos.140163

**Published:** 2015-01-14

**Authors:** Colin Palmer, Mark T. Young

**Affiliations:** 1 Ocean and Earth Science, National Oceanography Centre, University of Southampton, Southampton SO14 3ZH, UK; 2 School of Earth Sciences, University of Bristol, Bristol BS8 1RJ, UK; 3 Grant Institute, School of GeoSciences, University of Edinburgh, The King's Buildings, Edinburgh EH9 3FE, UK

**Keywords:** drag reduction, flow separation, fluid dynamics, Mosasauridae, riblets, shark

## Abstract

Living in water imposes severe constraints on the evolution of the vertebrate body. As a result of these constraints, numerous extant and extinct aquatic vertebrate groups evolved convergent osteological and soft-tissue adaptations. However, one important suite of adaptations is still poorly understood: dermal cover morphologies and how they influence surface fluid dynamics. This is especially true for fossil aquatic vertebrates where the soft tissue of the dermis is rarely preserved. Recent studies have suggested that the keeled scales of mosasaurids (pelagic lizards that lived during the Late Cretaceous) aided in surface frictional drag reduction in a manner analogous to the riblets on shark placoid scales. However, here we demonstrate that mosasaurid scales were over an order of magnitude too large to have this effect. More likely they increased the frictional drag of the body and may have played a role in controlling flow separation by acting as surface roughness that turbulated the boundary layer. Such a role could have reduced pressure drag and enhanced manoeuvrability. We caution those studying fossil aquatic vertebrates from positing the presence of surface drag reducing morphologies, because as we show herein, to be effective such features need to have a spacing of approximately 0.1 mm or less.

## 2. Introduction

Secondarily aquatic and fully marine amniotes convergently evolved similar osteological and soft-tissue adaptations as a result of the constraints imposed by living in water. Large-scale macroevolutionary trends such as tail flukes, limbs modified into paddles or flippers, and extra-renal mechanisms for salt excretion independently evolved in numerous marine amniote clades, with one or more found in: cetaceans, pinnipeds, turtles, birds, ichthyosaurs, sauropterygians, metriorhynchid crocodylomorphs and mosasaurid lizards (e.g. [1–16], and references therein).

Understandably, other adaptations such as dermal cover and its impact on hydrodynamics are less well studied and understood as only exceptionally well-preserved fossils retain skin covering [14,17–19] or the underlying dermal fibres [17,19,20,21]. Moreover, recent studies on shark placoid scales suggest that their primary benefit is not only surface drag reduction as commonly thought, but also (and perhaps more importantly) controlling flow separation and thus reducing pressure drag [22,23].

Here, we give an overview of shark scale hydrodynamics, the drag reduction due to their surface geometry and the flow control due to passive bristling. We then compare the potential drag reducing effect of the scale morphologies of the fossil pelagic vertebrate clade Mosasauridae, to those of sharks.

Recently, it has been suggested that the keeled scales of mosasaurids had a frictional drag reducing hydrodynamic function analogous to that of shark scales [18,19]. Here, we show that *contra* Lindgren *et al.* [18,19], it is highly unlikely that mosasaurid scales had any frictional drag reduction effects; in fact these keeled scales almost certainly increased the frictional drag compared with a smooth surface. We posit, based on research on shark scales, that if the mosasaurid scale roughness had any hydrodynamic function, it may have been beneficial in controlling flow separation, reducing pressure drag and possibly increasing manoeuvrability.

## 3. Surface fluid dynamics and frictional drag reduction

When fluid flows over a solid surface, there is a velocity gradient across the flow. At the immediate surface, the flow velocity is zero (the so-called no slip condition), but moving away from the surface the velocity tends asymptotically to that of the free stream. This velocity gradient results in shear stresses within the fluid, which are manifest as a horizontal force—the frictional drag. The region of the velocity gradient is called the boundary layer, and depending on the flow conditions, the flow can be either laminar or turbulent. The frictional resistance is reduced in regions of laminar flow, but this type of flow is unstable and only occurs over very small areas of the body surface of animals the size of sharks. For these animals, the predominant flow regime is turbulent.

Generally speaking, the frictional drag varies with the surface roughness, with the smoothest surfaces having the lowest resistance. However, at a very small scale, this may change and some specific forms of surface roughness can actually cause a reduction in frictional resistance. This happens because close to the surface in the viscous sub-layer, turbulent flow exhibits a somewhat regular steak pattern of longitudinal vorticity [24]. This provides the momentum transport through the sub-layer, and micro-turbulence or micro-bursts of fluid dissipate energy and thus increase the shear stress between the fluid and the surface of the body moving within it, so increasing the frictional drag. However, theoretical and experimental work has shown conclusively that the application of small, longitudinal ridges to the surface (commonly referred to as riblets) can bring a degree of order to this flow regime by channelling some of this turbulence and consequently reducing the frictional drag (reviewed in [25,26]).

Experiments have shown that reductions of friction drag by up to 10% are possible with carefully designed riblets [26,27]. These riblets need to take the form of continuous, narrow, parallel channels aligned with the flow, which are typically less than 0.1 mm wide and have somewhat less depth. The most effective shape has walls made from very thin vertical plates with sharp upper edges, which are so delicate as to be impractical. More practical shapes, which have a trapezoidal or semi-circular trough-like cross section are less effective but nonetheless can reduce drag by 6 or 7%. Materials with these geometries have been investigated for applications on aircraft and ships, with promising results [28]. In practice, the total frictional resistance reduction does not reach that demonstrated in the laboratory, but 3–5% seems to be achievable [26,28,29].

## 4. Drag reduction morphologies in sharks

The rough skin of sharks is caused by the dermis being covered by small placoid scales, also referred to as dermal denticles. These scales vary in size and shape, both between different shark species and across the skin of individuals, depending upon their location on the body [27,30]. Furthermore, faster swimming sharks have smaller placoid scales than slower swimming species [24]. Positional variation includes the degree to which the scales have surface grooves and the flexibility of their attachment to the dermis [24].

The manufactured riblets described above are morphologically very different from the geometry of placoid scales, but experiments have been conducted on shorter riblets arranged in a staggered layout that bear a closer resemblance to the arrangement of shark scales [25]. This arrangement was not as effective as thin parallel channels, but nonetheless produced a drag reduction of around 7%. Bechert *et al.* [25] also built models of placoid scales, which they tested in an ingenious oil-filled testing tank, allowing them to make models approximately 100 times larger than the actual scales while retaining Reynolds number equivalence. Comparing these results to those of the blade rib surfaces, Bechert *et al*. [25, p. 163] concluded: ‘even with a detailed and compliant shark skin replica, we did not find any striking effect, at least in as far as shear stress reduction is concerned. Actually, our synthetic two-dimensional blade rib surfaces perform significantly better than our ambitious shark-skin replica’.

One intriguing possibility is that some degree of randomness in the arrangement of the ribs might be beneficial. Small-scale roughness in the form of V-shaped protrusions has been reported to either increase or decrease drag, depending on their arrangement [31]. A random arrangement was found to decrease frictional drag by 10%, whereas the same shapes arranged in a regular pattern increased frictional drag by 20% [31]. That said, no verification or further development of this apparently important result is reported in the literature, which is perplexing given the potential commercial value of such measures. Indeed, other studies have been unable to reproduce this result [32,33]. However, research on seal fur (which is described as being hydrodynamically equivalent to somewhat random riblets) found drag reduction of approximately 12%, greater than the 7% found for conventional, regular riblets [34]. While inconclusive, taken together these results hold out the tantalizing possibility that randomness in the arrangement of short, natural riblets may be a source of additional, currently unquantified, frictional drag reduction.

Less speculatively, Büttner & Schulz [35, p. 2] reviewed the literature and concluded: ‘It is still not clear if the riblet-like surface on shark skin actually reduces turbulent viscous [frictional] drag. However, the technical analogy [parallel channel riblets] does reduce skin friction’.

More recently, Oeffner & Lauder [22] conducted experiments with actual shark skin, and Wen *et al*. [36] used high fidelity manufactured shark skin applied to an oscillating hydrofoil. Their results indicate that the shark skin surfaces not only reduced frictional drag, but that foils coated with shark skin also generated more thrust. This latter effect appears to be a result of reduced separation and increased leading edge vorticity, meaning that the placoid scales are acting both as friction reducing riblet surfaces and separation suppressing vortex generators. This latter effect could equally well have been achieved by another form of roughness as it is well established that vortex generation caused by surface roughness can delay flow separation.

Overall, these studies suggest that shark skin may well only have a small frictional drag reduction effect. And as Lang *et al.* [23, p. 2] note: ‘there still exists debate as to the dominant source of drag on swimming sharks (skin friction may be negligible compared to pressure and induced drag)’. If this is so, the effect on total drag of a small reduction in frictional drag may be very small indeed.

This is not to say that the highly derived shape of placoid scales is without any hydrodynamic function. They can be effective in delaying flow separation and thus reducing pressure (or form) drag, particularly where they are flexibly attached and can bristle [23,37]. As Lang *et al*. [23, p. 8] note: ‘these embedded vortices, analogous to dimples acting on a golf ball, may work as a boundary layer control mechanism to passively delay, or even prevent, flow separation, thereby reducing form drag’ and Lang *et al*. [37, p. 212]: ‘the findings that the most flexible scales are found on the flank of the body and downstream of the gills corroborate the hypothesis that these scales are working to control flow separation’. The flanks of the animal are where large adverse pressure gradients (a cause of flow separation) occur as the body flexes during swimming. Indeed, it may well be that the primary benefit of placoid scales is flow control that maximizes the animal's manoeuvrability and thrust production [22], rather than drag reduction in steady, straight line swimming.

## 5. Possible drag reduction morphologies in Mosasauridae

Mosasaurids were secondarily marine squamates (‘lizards’) that became the dominant marine reptile group in the Late Cretaceous, between 98 and 66 Ma [13,14]. They achieved a global distribution [38] and had a diverse array of trophic specializations [39–42]. Their adaptations to a marine existence were extensive, including: a hypocercal tail, hydrofoil-like limbs and putatively drag reducing scales [8,12–14,18,19,42].

All of the considerations mentioned above regarding drag reduction could apply equally well to mosasaurids. However, there is just one problem, that of scale. For maximum efficiency, riblets have to have a spacing of about 0.1 mm or less, and consequently the placoid scales of large sharks (e.g. the great hammerhead) are only around 0.2–0.3 mm long [28]. Calculations using the formulae of Büttner & Schulz [35] show that the optimum riblet spacing for a 1 m long animal swimming at 4 m s^−1^ is 0.10 mm, while for a 10 m individual at the same speed the size only increases to 0.12 mm. If such a large animal were to swim at 15 m s^−1^, the optimum size would reduce to 0.04 mm. It is clear therefore that the spacing of the riblets for all shark and mosasaurid-type animals must be of the order of 0.1 mm or less.

By contrast, scales from multiple mosasaurid genera (*Ectenosaurus*, *Plotosaurus* and *Tylosaurus*) were at least 2 ×2 mm [18,19]. This is more than an order of magnitude larger than those of large, fast sharks (sharks which have riblet spacing within the 0.1 mm window necessary for drag reduction).

While scale size was too large, what about riblet/keel separation? Interestingly, *Platecarpus tympaniticus* scales from across the body lack a keel [13], while *Ectenosaurus clidastoides* and *Tylosaurus proriger* have a single midline keel per scale [19,43]. Therefore, in these latter two species, there are macroscopic scales with a single keel (or riblet), thus the keels between the scales would have been widely separated. This is in contrast to shark placoid scales that are generally microscopic and have multiple riblets, typically ranging from three to eight, on a single scale [25]. The only mosasaurid suggested to have multiple keels per scale is *Plotosaurus bennisoni* [18]. Although preservation is incomplete, it seems as though three keels are restricted to the centre of the scale in *P. bennisoni* [18], and that the spacing between these ridges is approximately 0.3 mm (J. Lindgren 2014, personal communication), which is comparable to that of a modern tiger shark. However, Lindgren *et al*. [19] suggested that these structures may in fact be support sculpturing from the epidermis and not keels/riblets.

Experimental work on riblets has shown that as size increases beyond the optimum, the drag reduction effect is quickly lost and drag starts to increase as the flow responds to the larger riblets as simply surface roughness [28,35,44]. Consequently, the size of the mosasaurid scales and riblet spacing is such that, *contra* Lindgren *et al.* [18], it is highly unlikely that they had any drag reduction effect resulting from effects analogous to shark scale riblets; indeed they most likely increased the drag compared with a smooth surface. The resulting turbulence generation due to the roughness of the scales could have been beneficial in controlling flow separation, with the associated benefits described above, but this could be achieved by any surface roughness, not a specific scale morphology (for example, if the raised distal margins of *Platecarpus* scales are not artefactual—see fig. 5 in [13]—then this too could generate turbulence). Note that there would only have been a significant increase in thrust if the keeled scales are on the thrust-producing hypocercal tail.

## 6. Conclusion

Herein, we demonstrate that mosasaurid scales (and keel spacing) were too large for them to have had a frictional drag reduction effect analogous to that attributed to shark placoid scale riblets, despite their appearing to become smaller in more derived/better aquatically adapted forms (J. Lindgren 2014, personal communication). Moreover, mosasaurid scales most likely increased the frictional drag. Consequently, we need to look elsewhere for the possible reasons for the development of this scale morphology. One hydrodynamic possibility was controlling flow separation.

Currently, the skin surface of other Mesozoic reptilian clades is largely unknown, although it is possible that ichthyosaurs had scaleless, smooth skin [20]. It should be noted that other hypotheses have been put forward for both mosasaurid keeled scales and ichthyosaur ‘rippled’ skin, such as limiting ectoparasite attachment [18,19]. This hypothesis has also been proposed for the grooves on shark placoid scales, but again these morphologies are at least an order of magnitude smaller than those mosasaurid scales [25,45,46]. Other adaptations, from thermoregulation to crypsis, have also been posited for the presence of keeled scales [18,47]. While these grooved-ridged skin surface morphologies could have had multiple functions, we argue caution when postulating hydrodynamic functions for extinct aquatic vertebrates.
